# Editorial: Rural disability and community participation

**DOI:** 10.3389/fresc.2022.1049578

**Published:** 2022-10-17

**Authors:** Catherine Ipsen, Jean P. Hall, John Lui

**Affiliations:** ^1^ University of Montana, Missoula, MT, United States; ^2^ University of Kansas, Lawrence, KS, United States; ^3^ University of Wisconsin–Stout, Menomonie, WI United States

**Keywords:** rural, disability, participation, community living, disparities

**Editorial on the Research Topic** Rural disability and community participation by Ipsen C, Hall JP and Lui J. (2022) Front. Rehabilit. Sci. 3: 1049578. doi: 10.3389/fresc.2022.1049578

The barriers, needs, and opportunities of people with disabilities living in rural communities can be very different than those living in urban areas. This situation is problematic when concerns, approaches, and policies are defined through an urban lens, and overlook or disregard rural community values, systems, and decision-making. Rural research, innovation, and evidence-based practices are essential for understanding and addressing disability issues within the rural context.

The Rural Disability and Community Participation research topic contributes to the current science of disability and rehabilitation from a rural perspective. We know that rural people with disabilities experience a variety of social and economic disparities that affect and limit community inclusion and participation. These inequalities include access to education, employment, transportation, health care, and community services. Because people with disabilities and underserved rural populations are both considered health disparity groups, the intersection of these identities introduces compounded disadvantage.

For this special topic, we reached out to rural disability researchers across the US and internationally. We received nine articles that addressed disability disparities, community access, and infrastructure from rural perspectives.

The first three articles focused on the experience of rural disability and factors that predict or are associated with elevated rural disability rates. Ipsen, Ward and Myers examined 27 waves of U.S. National Longitudinal Survey of Youth data to explore how environmental factors over the life course, such as occupations, injuries, access to health insurance, and living in a rural location, predicted mobility disability at age 40 and age 50 Ipsen et al. (2022). After controlling for both socio-demographic characteristics and life events, they reported living in a rural community increased the odds of mobility impairment. These findings reinforce the value of consistent and adequate health care access and exploring additional rural community factors that contribute to disability.

Mashinchi, Hicks, Leopold, Greiman, / Ipsen used American Community Survey (ACS) 5-year estimates to conduct geographic analyses of disability rates for American Indians/Alaskan Natives (AI/ANs) living in metropolitan, micropolitan, and noncore counties Mashinchi et al. (2022). Generally, these data aligned with past studies indicating greater disability rates among AI/ANs compared to Whites. However, differences in disability rates between AI/AN and White racial groups were no longer present when comparing counties with a significant AI/AN presence (≥5% of the county population is AI/AN). The authors highlight the potential protective factors offered by sense of belongingness and cultural fit.

von Reichert explored disability from a household context using the 2015–2019 ACS Public Use Microdata Sample Von Reichert (2022). In addition to describing an innovative method for classifying cases across the rural-urban continuum, von Reichert found that living alone was more prevalent for people with disabilities living in rural areas and multigenerational households with disability were more common in large cities.

Other articles focused on rural participation from the lens of access to services in the community. Myers, Ipsen, and Standley explored 2017 National Household Travel Survey data to explore rural and urban differences in transportation patterns for people with travel-limiting disabilities Myers et al. (2022). Their paper examined differences between rural and urban drivers and non-drivers, types of transportation, and how adults with disabilities decide if they will give up driving. The results illustrate significant disparities in transportation options and offer policy and community insights for improving rural transportation systems.

Gimm and Ipsen used data from the National Survey on Health and Disability to explore rural and urban differences in both unmet and perceived need for acute and preventive services Gimm and Ipsen (2022). They found similar rates of unmet need across respondents from rural and urban locations, but significant differences in perceived need for preventive services. Specifically, rural people with disabilities reported not needing dental and mental health counseling at significantly higher rates than their urban counterparts. These differences highlight the impact of community norms and expectations in terms of rural health disparities.

Sage, Standley, and Mashinchi examined the rights of both disabled people and home-based personal care workers through the historical progression of federal policies and support of personal assistance services (PAS; Sage et al. (2022). Their paper explored the current and future implications on rural communities and highlighted the complex social justice issues that arise when trying to elevate the needs of different groups. This contextual work was complemented by a second paper by the same authors that surveyed consumers of PAS in five states to explore satisfaction with services and community participation outcomes among metro and non-metro respondents Sage et al. (2022). Overall, there were few rural and urban differences, and more research is needed to understand features of effective PAS delivery.

The final two articles focus on rural community infrastructure and strategies for measuring it. Seekins, Traci, and Hicks provided a process and strategy for assessing the accessibility of community space using Google Earth and Google Street View Seekins et al. (2022). Using existing Google imagery and an observation rating protocol, they assessed a total of 47 rural and urban communities and a combined 79 miles of community pathways to derive Community Access Scores (CAS) and Rule of Proportional Participation (RPP) rates. In general, rural communities had lower CAS scores and lower RPP rates, indicating participation limitations in both opportunities and use.

Finally, Hicks, Traci, and Korb compare disability simulations and I2audits for creating public awareness of access issues Hicks et al. (2022). Disability simulations ask participants to role-play different disability experiences, such as traveling in a wheelchair or wearing a blindfold. I2Audits involve a “shared discovery” of public access features with an interdisciplinary team of disability, public health, and public planning stakeholders. The authors conducted qualitative interviews with stakeholders who had participated in these strategies and concluded the I2Audits reduced feelings of stigmatization and provide opportunity for meaningful community dialogue.

Overall, rural disability research is varied in focus and approach. What is common across themes is the persistent disparity of health outcomes, lack of available resources, and feelings of uncertainty pervading an increasingly complex rural environment. Articles call for additional research to develop strategies to empower people with disabilities to meaningfully participate in their rural communities.

